# Delirium and Impact on Physical Function in Hospitalized Persons Living With Dementia

**DOI:** 10.1093/geroni/igaf122.724

**Published:** 2025-12-31

**Authors:** Ashley Kuzmik

**Affiliations:** Alzheimer’s Association, Chicago, Illinois, United States

## Abstract

Delirium is a common occurrence among hospitalized older adults living with dementia, and it poses significant risks to their health and wellbeing. The presence of delirium among older adults, especially those with dementia, is associated with low levels of physical function while hospitalized. The aim of this study was to investigate whether demographic characteristics (i.e., age, sex, and race) moderate any differences in delirium severity as predictors of physical function in 351 hospitalized persons living with dementia. Age and sex but not race had significant moderating effects on the relationship between delirium severity and physical function (β = 2.22; p = 0.02 and β = 1.34; p = 0.04, respectively). Notably, older adults aged ≥ 85 years with higher levels of delirium severity had lower levels of physical function compared to older adults aged 65-84 years . Males with higher levels of delirium severity had lower levels of physical function compared to females. These findings suggest that future clinical interventions, such as use of function focused care, be tailored to address the unique needs and challenges faced by older males particularly to best address delirium and optimize physical function when hospitalized.

